# Supplementary material for: Iterative improvement in the automatic modular design of robot swarms
Source: PeerJ Comput Sci. 2020 Dec 7;6:e322. doi: 10.7717/peerj-cs.322 (PMC7924708; doi:10.7717/peerj-cs.322)
Supplement: Supplemental Information 3 [file peerj-cs-06-322-s003.zip › argos3/doc/api/standalone/a00551_source.html]

ARGoS: plugins/robots/mini-quadrotor/simulator/miniquadrotor\_entity.h Source File


- Main Page
- Related Pages
- Namespaces
- Classes
- Files

- File List
- File Members

# plugins/robots/mini-quadrotor/simulator/miniquadrotor\_entity.h

Go to the documentation of this file.

```
00001 
00007 #ifndef MINIQUADROTOR_ENTITY_H
00008 #define MINIQUADROTOR_ENTITY_H
00009 
00010 namespace argos {
00011    class CControllableEntity;
00012    class CEmbodiedEntity;
00013    class CRotorEquippedEntity;
00014 }
00015 
00016 #include <argos3/core/simulator/entity/composable_entity.h>
00017 #include <argos3/plugins/robots/mini-quadrotor/control_interface/ci_miniquadrotor_trajectory_actuator.h>
00018 
00019 namespace argos {
00020 
00021    class CMiniQuadrotorEntity : public CComposableEntity {
00022 
00023    public:
00024 
00025       ENABLE_VTABLE();
00026 
00027    public:
00028 
00029       CMiniQuadrotorEntity();
00030 
00031       CMiniQuadrotorEntity(const std::string& str_id,
00032                      const std::string& str_controller_id,
00033                      const CVector3& c_position = CVector3(),
00034                      const CQuaternion& c_orientation = CQuaternion());
00035       
00036       virtual void Init(TConfigurationNode& t_tree);
00037 
00038       inline CControllableEntity& GetControllableEntity() {
00039          return *m_pcControllableEntity;
00040       }
00041       
00042       inline CEmbodiedEntity& GetEmbodiedEntity() {
00043          return *m_pcEmbodiedEntity;
00044       }
00045 
00046       inline CRotorEquippedEntity& GetRotorEquippedEntity() {
00047          return *m_pcRotorEquippedEntity;
00048       }
00049       
00050       virtual std::string GetTypeDescription() const {
00051          return "mini-bot";
00052       }
00053 
00054       inline const CCI_MiniQuadrotorTrajectoryActuator::SWaypoint& GetDesiredWaypoint() const {
00055          return m_sDesiredWaypoint;
00056       }
00057 
00058       inline void SetDesiredWaypoint(CCI_MiniQuadrotorTrajectoryActuator::SWaypoint& s_waypoint) {
00059          m_sDesiredWaypoint = s_waypoint;
00060       }
00061 
00062    private:
00063 
00064       CControllableEntity*                           m_pcControllableEntity;
00065       CEmbodiedEntity*                               m_pcEmbodiedEntity;
00066       CRotorEquippedEntity*                          m_pcRotorEquippedEntity;
00067       CCI_MiniQuadrotorTrajectoryActuator::SWaypoint m_sDesiredWaypoint;
00068    };
00069 
00070 }
00071 
00072 #endif
```

---

Generated on 10 Jul 2018 for ARGoS by 
 1.6.1
